# The use of electronic patient records for medical research: conflicts and contradictions

**DOI:** 10.1186/s12913-015-0783-6

**Published:** 2015-03-29

**Authors:** Fiona Stevenson

**Affiliations:** Department of Primary Care and Population Sciences, UCL, London, UK

**Keywords:** England, Use of patient records for research, Implementation, Data linkage, Information governance, Consent, General practice, Qualitative research

## Abstract

**Background:**

The use of electronic patient records for medical research is extremely topical. The Clinical Practice Research Datalink (CRPD), the English NHS observational data and interventional research service, was launched in April 2012. The CPRD has access to, and facilities to link, many healthcare related datasets. The CPRD is partially based on learning from the Health Research Support Service (HRSS), which was used to test the technical and practical aspects of downloading and linking electronic patient records for research. Questions around the feasibility and acceptability of implementing and integrating the processes necessary to enable electronic patient records to be used for the purposes of research remain.

**Methods:**

Focus groups and interviews were conducted with a total of 50 patients and 7 staff from the two English GP practices involved in piloting the HRSS, supplemented with 11 interviews with key stakeholders. Emergent themes were mapped on to the constructs of normalization process theory (NPT) to consider the ways in which sense was made of the work of implementing and integrating the HRSS.

**Results:**

The NPT analysis demonstrated a lack of commitment to, and engagement with, the HRSS on the part of patients, whilst the commitment of doctors and practice staff was to some extent mitigated by concerns about issues of governance and consent, particularly in relation to downloading electronic patient records with associated identifiers.

**Conclusions:**

Although the CPRD is presented as a benign, bureaucratic process, perceptions by patients and staff of inherent contradictions with centrally held values of information governance and consent in downloading and linking electronic patient records for research remains a barrier to implementation. It is likely that conclusions reached about the problems of balancing the contradictions inherent in sharing what can be perceived as a private resource for the public good are globally transferrable.

## Background

The use of electronic patient records for medical research is extremely topical. The Clinical Practice Research Datalink (CRPD), the English NHS observational data and interventional research service, was launched in April 2012. The CPRD has access, and facilities to link, to many health and social care related datasets. The CPRD is ideologically driven leaving questions around the acceptability of implementing and integrating the necessary processes to enable electronic patient records to be used for the purposes of research unresolved. This paper examines the idea that although the CPRD is presented as a benign and bureaucratic imperative which will produce benefits at both individual and societal levels, the processes involved in the collection of electronic patient records for research contradict with centrally held values of information governance and consent causing problems for implementation.

One by-product of the universal health care system in the UK (the NHS) is the quantity of longitudinal health data. The almost universal use of electronic patient records in primary care in particular provides the potential to address new research questions using these data, particularly when linked to data from other sources such as social care.

There is a strong political imperative to use electronic patient records for research. The perceived value of their utilisation was made clear in the publication of The Plan for Growth [1], while the recent update to the NHS constitution [2] presents research as a ‘core’ activity of the NHS making the link between the provision of NHS services and research explicit.

However, alongside the rhetoric of the value to the UK of the use of electronic patient records for research, recognition of public and professional disquiet has led to delays in implementation of the necessary systems in primary care.

Concerns have been raised about the commodification of patient records [3], the use of records for purposes other than they were originally collected and potential problems in relation to the presumed accuracy of original data [4]. Although, views about sharing data from medical records are generally altruistic [5], concerns have been expressed about the use of identifiable data [6] and sharing of data with commercial agencies [7-9]. In summary, the belief that an individual has a ‘natural right to privacy’ appears to be (precariously) balanced with a genuine commitment to support medical research [10]. Concerns about balancing privacy against the public good are at the heart of the decision to be involved in any research; arguably a particular problem associated with the use of patient records for research is that there is no direct, visible link between the provision of data and the research for which it is used.

This paper explores the likely challenges to implementation of the CPRD. Following a brief outline of the background to CPRD, normalization process theory (NPT) is used as a framework within which to explore data concerned with downloading electronic patient records from GP practices.

### Background and development of CPRD

The stated aim of the CPRD is to maximise the way anonymised NHS clinical data can be linked to enable observational research and deliver research outputs that are beneficial to improving and safeguarding public health (http://www.cprd.com/intro.asp).

Overall coverage of existing databases used for health services and epidemiological research (General Practice Research Database (GPRD), The Health Improvement Network (THIN), QRESEARCH, IMS Mediplus system) is estimated to be only about 20% of NHS patients and focuses on primary healthcare data, with limited linkage to other records. The CPRD aims to gain nationwide primary care data and crucially linkage of data across a range of settings.

Currently the main primary care database held by CPRD is known as GOLD (formerly GPRD). GOLD contains the anonymised, longitudinal medical records of patients registered with contributing primary care practices across the UK. The GOLD database covers approximately 8.8% of the UK population, including practices in England, Northern Ireland, Scotland and Wales. As of September 2014 there were 684 GP practices and 13.58 M acceptable (research quality) patients in GOLD, of which 5.69 M are active (still alive and registered with the GP practice). Data has been collected from GP practices since 1987. Historically, less than 0.5% of patients from these practices have opted out of their records being included.

A nationwide rollout of the CPRD was due to begin in the Spring 2014 but was postponed following opposition from senior GPs, privacy campaigners and online campaign groups (such as 38 Degrees) [11]. It was re-launched in October 2014, initially in six Clinical Commissioning Groups (local GP-led organisations), with a view to nationwide rollout [12,13].

The CPRD is partially based on a pilot programme; the Health Research Support Service (HRSS). The HRSS sought to extract electronic records from across health and social care sectors and to transfer them together with associated identifiers (such as name, address, date of birth) to a designated “safe haven” (in which information is processed independently of both the data source and the researcher that requires the data). Extracted data were linked to census data, public health data and mortality data.

One of the key questions addressed by the HRSS pilot in primary care was the feasibility of seeking consent for electronic records, together with associated identifiers, to be downloaded into the “safe haven”. As part of the HRSS pilot all patients (with the exception of students and homeless people in one practice) in two general practices were contacted in writing informing them of their right to opt out of their electronic medical records being downloaded for possible use in research.

The use of an opt out as opposed to an opt in is particularly controversial. The reasoning behind the use of an opt is that it increases the numbers participating as it does not require people who have no objection or are neutral about participation to act. However the other side to this is that there is no way of being sure that those people who do not opt out are happy for their records to be used. Such debates are currently being discussed in relation to European Privacy Legislation (http://ec.europa.eu/justice/data-protection/), which in the event of a requirement to have an opt in for the use of data could lead to an enforced change in the operating practices of the CPRD.

### The problem

This paper considers the idea that the CPRD is presented as a benign and bureaucratic imperative which will provide benefits at both the individual and societal level, yet evidence from a qualitative evaluation of the HRSS pilot (learning from which the CPRD is partially based) indicate that the processes involved in making electronic patient records available for research may contradict with other centrally held values, in particular information governance and consent.

## Methods

The HRSS pilot sought to extract electronic medical records from two GP practices. Ethical approval was obtained from North West London REC 1, REC reference number: 10/H0722/26. Following research and development approvals a qualitative evaluation was conducted in both practices. The evaluation team played no part in the design or implementation of the HRSS pilot.

### Interviews with key stakeholders outside of the practices

Factors that influence the translation of an innovation into routine practice arise at the individual, organisational and wider levels of healthcare systems and interact in complex and variable ways [14]. In order to provide contextual information, interviews were conducted with people from outside the practices with a known interest in the use of electronic patient records for research. This was a purposive sample with participants recruited following non-participant observations of meetings concerning the use of electronic patient records for research supplemented by direct approaches to key experts.

A semi structured interview schedule was used that focused on views of the use of electronic patient records for research in general and the HRSS in particular. Participants were asked to consider how the HRSS fitted with other work on using electronic patient records for research, barriers and facilitators to the HRSS and the key principles that should inform the implementation of the HRSS pilot project. Interviews typically lasted for about 60 minutes.

### Group discussions and interviews with patients

Each participating practice selected a random sample of 200 patients who had opted out of their electronic medical records being downloaded for the HRSS pilot and 200 patients who had not. Letters sent on practice headed notepaper invited patients to take part in their choice of an interview or group discussion. Forms noting interest were returned directly to the research team using a pre-paid envelope.

Interviews and group discussions took place within local community venues. All participants received an information sheet and provided written consent. A £20 voucher was offered as a token of appreciation. A brief overview of the HRSS pilot study was given prior to beginning each group discussion or interview.

Group discussions were facilitated by two researchers and typically involved between 5 and 8 patient participants. They started with an interactive task involving working in groups and writing on a flipchart what they knew/understood about the HRSS pilot prior to a re-cap by the researchers and their views and opinions of it before and after the re-cap. This formed the basis for discussion. Groups also explored attitudes to sharing data and to consent and views on any future roll out of the HRSS. Patients who were unable to come to a group were individually interviewed about the same issues but without the interactive task. Relevant topics were incorporated into the topic guide. Sessions typically lasted for between 50 and 70 minutes.

### Interviews with practice staff

Staff were asked for their understanding of the HRSS pilot, how they felt the pilot had worked in practice, and their thoughts on the use of an “opt out” and the future roll out of the HRSS. Interviews typically lasted for between 20 and 30 minutes.

### Data analysis

Interview and group discussion data were recorded digitally and fully transcribed, with the exception of three stakeholder interviews from which notes were taken and written up immediately following the interview.

All interviews were analysed thematically with themes independently developed by three researchers and presented and discussed in steering groups meetings. Following MacFarlane and O’Reilly-de Brun [15], themes from interviews and focus groups with practice staff and patients were then mapped onto the constructs of the Normalization Process Theory (NPT). Normalization Process Theory (NPT) is concerned with the processes by which practices become routinely embedded in everyday life [16] and was used here as an organisational framework to explore patients’ and practice staff’s experiences and understandings of the processes involved in electronic patient records being included as part of the HRSS. Data from interviews with stakeholders from outside the practices provided contextual information to inform the ways in which people made sense of the work of implementing and integrating the HRSS pilot.

All names used are pseudonyms.

## Results

### Stakeholder interviews

Eleven interviews were conducted with people with backgrounds in academia, policy and medicine to ensure data from a range of perspectives.

### Patient participants

Of 800 patients approached, 79 (10%) indicated their willingness to participate in the evaluation and 50 finally participated, the majority of whom reported not opting out of the HRSS pilot project. Six focus groups and 17 interviews were conducted.

Patients were predominantly female and clustered at the older end of the age spectrum. Nine of the 50 patient participants indicated, without prompting, that they had either a current or former professional interest in healthcare or research.

### Staff participants

Interviews were conducted with all key staff members. In total, 6 interviews were conducted with 7 different staff members.

### The CPRD as a benign, bureaucratic imperative

Unsurprisingly key stakeholders from outside the practices were generally positive about the use of electronic patient records for research, describing the development of the HRSS as an invaluable resource for researchers and a unique opportunity to present the UK as a world leader in medical research.

This has to be the future for research in this country… there is such a wealth of knowledge locked up that we must have access to (S1)

It was suggested such a resource would support clinical innovation and strengthen evidence of effectiveness resulting in improvements in health outcomes, with drug safety particularly singled out.

There was however explicit discussion about the need to balance ‘public good’ against the risks of adversely affecting the trust people have in doctors and the medical system. The importance of strong governance procedures was stressed together with the need to communicate the fact that data loss or personal identification has never occurred as a result of using electronic records for research.

The HRSS pilot required people to opt out if they did not want their records to be used for research. Concerns were raised about the acceptability of using an ‘opt out’ as a proxy for consent. This was presented by one interviewee as a “political hot potato”, with another saying it was only possible because the HRSS was a pilot project.

Despite wholehearted support for the use of electronic patient records for research, both the necessity and feasibility of gaining the nationwide coverage sought by the HRSS was challenged, suggesting rather that statistical methods could be used to impute effects. Finally, concerns were raised that an increase in quantity of data would lead to problems with standardisation and data quality. These concerns were in some ways mitigated by the argument that the quality of UK medical data is generally high:

Our worse data is better than most other countries’ best data. we shouldn’t kick ourselves in the foot (S2)

#### The experience of the HRSS

Data from interviews and focus groups with patients and practice staff have been organised according to the four components of NPT; coherence (whether people understood the HRSS), cognitive participation (whether they were happy to participate), collective action (the work people had to do in relation to participation in the HRSS), and reflexive monitoring (comments on the future operation of the HRSS). Within this collective action was divided into four elements (1) *interactional workability* (2) *relational integration*, 3) *skill set workability,* and (4) *contextual integration.*

### Understanding the HRSS (coherence)

Focus groups and interviews indicated a number of misunderstandings about the HRSS and the processes involved suggesting a possible problem with coherence. There were four fundamental points of misunderstanding: (1) patients believed they had been selected (rather all patients in the practice were contacted) (2) patients did not understand they may be contacted about involvement in a research project on the basis of information from their medical record, (3) both patients and staff were unaware that data would not be anonymised prior to leaving the practice and (4) that participation required no action, action was only necessary to opt out. There was also confusion between the HRSS and the summary care record scheme on the part of patients and staff.

Patients had been sent information packs about the HRSS pilot, with staff informed in practice meetings. The information packs sent to patients were described as over complicated and unclear, and the accompanying letter vague. A number of patients did not recall receiving a letter about the HRSS which suggests neither the information received nor the associated decision making were memorable.

P1 We got a vague letter, didn’t we?

P2 I was going to say we got a letter a while ago. Obviously it got binned and I don’t remember what it said. (FG1 Practice 1)

Generally, it was thought the quantity of information provided was excessive, while practice staff, considering their practice population as a whole, expressed concerns about literacy and language difficulties.

Practice staff reported that the initial introduction to the HRSS was done in a busy practice meeting with insufficient time for discussion. It was only following a meeting once data had been readied for transfer staff felt they fully understood what was being asked of them and why. Moreover, although people in key roles in the practice developed a good understanding of the HRSS, this was not the case for people who were more peripheral.

I didn’t think this is a practice-wide project at all, to be honest; I think there’s just a few key people in the practice that knows what’s going on. I think if you went out to reception and said what’s HRSS they wouldn’t have a clue. (Staff Practice 1)

The use of opt out (as opposed to opt in) was a key feature of the HRSS. Views concerning the use of opt out differed, some people suggested that it was easy to miss the fact that you had to opt out, others said this was clear. Even where people stated they understood how they were expected to act they still appeared unclear about the implications of the process.

And I think, as Amelia was saying, it’s clarity of the whole situation about what this data is going to be used for… (FG 4 Practice 1)

In terms of NPT, there appeared to be a problem with coherence. However most patients reported they understood the HRSS following information provision as part of the qualitative evaluation, while practice staff reported understanding when information was provided just prior to records being downloaded. This suggests that, in terms of NPT, there is potential for coherence.

#### Commitment and engagement (cognitive participation)

Patients’ accounted for participation according to factors other than engagement with the HRSS. Accreditation from the NHS or practice was important, as one person put it:

I’m wary about it, but the fact that it has the… it’s under the auspices of the NHS rather than, if you like, Bloggs whatever; if it was Bloggs whatever… I wouldn’t do it. (FG 2 Practice 2)

Some patients associated participation with general support for research, or emanating from a feeling of social responsibility and the opportunity to ‘give something back’. Others said they did not see involvement as problematic, as they had nothing to hide.

When the letter came in, from what I remember, what registered was research, local doctor’s practice and I think, somewhere, there was an NHS logo and I thought, well it must be kosher and also I think it was probably from the angle of wanting to give something back. (FG2 Practice 2)

Finally, some patients said that just because it is possible to make patient records available for research cannot be seen as justification for handing over patients’ electronic medical data “ad lib in an identifiable manner” (FG 4 Practice 2).

For some practice staff the HRSS made perfect sense in terms of the most efficient use of a valuable resource.

So we’ve always, kind of, wanted to use data efficiently and been frustrated that the NHS doesn’t generally use data efficiently, so you know, it’s certainly ticked the box as far as what we believe should happen about the appropriate use of data (Staff Practice 2)

Commitment and engagement however appeared to be based on investment in the concept rather than necessarily trust in the processes used to implement the HRSS.

I think there’s a lot of trust that’s important in rolling this out, so the people who take it on trust that, yes, this is a safe, secure process and there’s a benefit worth taking any small risks there is of data breach. (Staff Practice 2)

Despite investment in the overall principle of the use of electronic patient records for research, concerns focused on two keys aspects; (i) the transfer of identifiable data in order to populate the databases and (ii) the use of opt out as a proxy for consent.

SM1: But when it was first sold to us we did get really excited about it initially because we thought we can’t believe that, in this day and age, there isn’t this facility already … to have information that researchers can tap into and to really develop some evidence based medicine … that was really exciting and we thought we should definitely be involved in that. So I guess that’s still there in the background, it’s just how the process to get that information is what we feel uncomfortable with. I still feel really comfortable with the principle of being involved in the research, but it’s just the process…

SM2: The opt-in or opt-out…. and if it was anonymised data I’d have no problem with it, but it’s not (Staff Practice 1)

In both practices a GP led involvement. Interestingly, one of them stated they would not proceed without the consent of the rest of the practice, thus despite their commitment to the concept of the HRSS their relationship with practice colleagues was paramount.

Because I’m not going to bully them, if colleagues aren’t comfortable with the model that’s been suggested, then it will go (Staff Practice 2)

In summary, there was a lack of commitment and engagement by patients, evidenced by the fact reasons for participation generally did not specifically refer to the HRSS. Among practice staff, despite commitment to the concept, concerns relating to governance and consent were seen to conflict and present a potential barrier to engagement.

#### The ‘work’ involved in participation (collective action)

The ‘work’ involved in participation in the HRSS can be divided into four aspects (i) the work patients did with practice staff and documentation when considering their participation in the HRSS (interactional workability), (ii) concerns about research governance and the HRSS and possible effects on relationships between patients and the practice (relational integration), (iii) the allocation of work associated with the HRSS (skill set workability) and (iv) the execution of protocols, policies and procedures in order to implement the HRSS (contextual integration).

### Patients ‘work’ (interactional workability)

Despite concerns about the quality and quantity of information sent, patients did not present the work involved in participation in the HRSS as particularly burdensome; although reports of not responding in time to opt out meaning records were included without consent indicates this process, in some cases at least, might have been experienced as problematic.

I know a lot of people who got the letter just put it to one side and thought oh, I’ll deal with… I’ll read that later. And then later’s too late, you find, you know, oh my God, it should have been back last week. I’m in and I don’t really want to be in; how do I get out? How do they get out? Is there an escape? Is there a mechanism for getting out if you do not want to be in there? (FG4 Practice 2)

GPs did not report any discussion about the HRSS with patients.

### The effects on relationships (relational integration)

Practice staff were concerned about releasing identifiable patient data and the associated responsibilities of information governance.

They [the practice] signed up to certain principles, one of which was about consent and confidentiality. So … to what extent is this project in conflict with what we said we’d sign up to… (Staff Practice 1)

Crucially concerns focused on their own practice, not the wider programme. Thus concerns were expressed about the removal of data from the control of the practice, with a query raised about why the HRSS was necessary if researchers would still have to contact the practice if they wished to directly involve patients in a research project.

we’ve handed over un-anonymised patient data to sources who aren’t directly involved in the patient care, which in terms of information governance is a bit of a big no-no really. ..we had a meeting last week with [GP lead] and he said that the researchers would interrogate that database, but then they would contact us to contact the patients. So it doesn’t seem like you’re really cutting out that… why don’t the researchers just contact us and we’ll tell them (Practice 1)

Questions were raised about why the whole record was taken instead of just the aspects necessary for particular research projects, with concerns expressed in particular about the lack of explicit consent from patients for downloading their electronic records.

### The allocation of work (skill set workability)

The main impact was on the practice staff responsible for preparing mailing lists, placing markers on the records of those who wanted to opt out of their records leaving the practice, and complying with the processes and timings involved in providing data for the HRSS pilot.

Both practices had an active patient participation group and each received a presentation on the HRSS from the implementation team. Each group was asked to nominate a representative to sit on the national HRSS patient participation group. One group failed to recruit a volunteer. The patient representative from the other practice only attended once and then resigned stating she did not feel she could contribute. This can be taken as a strong indicator of a lack of engagement by patients with the HRSS.

### Implementing the HRSS (contextual integration)

Patients’ knowledge and experience of conducting research was presented as a key factor in judgements as to whether or not to allow their data to be used to populate the HRSS. For some, previous knowledge and involvement in research meant they could see the value of an opt out as a proxy for consent. Others with the same background either in research or the health service, although acknowledging the potential value of the HRSS, opted out for fear their medical record would be recognised by other researchers.

In relation to policies and procedures, concerns focused on the fact there is no way of knowing if people receive a letter, and even if it is received if they understand it, yet records were included unless patients opted out. This concern was shared by patients and staff.

…I think really a lot of people have opted in by default (FG 2 Practice 2)

I’m quite uncomfortable with it [opt out] really, for me, just because all the research that we’ve ever done before has always been with the explicit consent of the patient (Staff Practice 1)

Anxiety was expressed about the possible adverse effects on computer systems when the download happened. The use of a computer programme for data cleansing, as opposed to a person who could identify individuals from the data, was however judged to be appropriated.

#### Reflections on the process (reflexive monitoring)

The data presented in this paper were collected prior to electronic patient records being downloaded. It is however important to note concerns were expressed by patients, practice staff and GPs that data protection may lessen as time goes on, this, together with concerns about the possible future sale of data, formed the backdrop to decisions made about participation in the HRSS.

…once it’s held, you know as well as I do what’s to stop, in the fullness of time, insurance companies coming up and saying oh, we’ll give you… buying data (FG4 Practice 2)

## Discussion

The CPRD combines learning from the GPRD (an existing database of electronic patient records used for research) and the HRSS pilot. This paper argues that the CPRD is associated with an ideology that it is difficult to disagree with; namely that electronic patient records should be used to inform research to improve patient health. Learning from the evaluation of the HRSS pilot indicates that although it may be technically possible to implement the CPRD, problems identified following an analysis organised according to the constructs of NPT suggest the planned nationwide rollout may prove problematic.

Existing databases of electronic patient records used for research (THIN, GPRD, QResearch) operate without apparent concerns from the patient population. A crucial difference however is that with the HRSS (and now CPRD) anonymisation takes place after records are downloaded into a ‘safe haven’ in order to facilitate the linking of data from a range of sources. In relation to the CPRD GOLD approximately 75% of the contributing practices in England (the CPRD currently only draws data from practices in England), or roughly 55% of all practices in the database are available for linkage. This suggests that up to 25% of the practices previously contributing to GPRD have not consented to participate in the linkage scheme (involving a change at the point of anonymisation), potentially indicating resistance even from those who have previously been prepared to provide data from patient records for the purposes of research .

The HRSS was used to pilot the technical feasibility of downloading electronic patient records into a safe haven for use in research. Analysis organised according to the constructs of NPT was used to show how the HRSS pilot project (upon which the CPRD is partially based) was understood once explained as part of the research, demonstrating the potential for widespread understanding of the CPRD. There was, however, a lack of commitment to, and engagement with, the HRSS on the part of patients, whilst the commitment of doctors and practice staff was to some extent mitigated by concerns about information governance and consent, focusing in particular on downloading electronic patient records with their associated identifiers. Use of an opt out as a proxy for consent was experienced as problematic for staff and patients alike, with some patients struggled with the work involved in opting out. Concerns were also expressed about decontextualisation of data and a lack of control over its use and the ways in which electronic patient data might be used in the future, particularly in relation to potential commercial use of data, a concern expressed more generally by, for example, online campaign groups such as 38 Degrees. In summary, arguments for the benefits of CPRD are generally positioned at the national and even global level, yet this research demonstrates that participants’ concerns remain at the individual and practice level.

In relation to the balance between privacy and the public good the findings indicated that although the idea of using patient records for research was accepted as worthwhile and useful, concerns were experienced in relation to the practical issues of information governance and consent. This research finding is in keeping with the reasons given for the six month delay to the rollout of the CPRD; which was said to allow time for a publicity campaign to explain the scheme and ensure individuals are aware of their right to opt out [11]. Although, as noted earlier, European Privacy Legislation (http://ec.europa.eu/justice/data-protection/), could result in a requirement for an opt in for the use of data leading to an enforced change in the operating practices of the CPRD.

The fact that key stakeholders from outside the two research practices emphasised the importance of engagement with patients and practices and also expressed reservations about the use of an opt out as a proxy for consent, provides additional impetuous for taking account of the issues identified by the NPT analysis presented here.

It should be noted that the practices in which the HRSS was rolled out were research practices with an enthusiastic GP supporting the HRSS and patients who were familiar with medical research. Thus it is particularly important to take account of the issues raised in relation to the likelihood of success of the planned future roll out of the CPRD across all general practices.

## Conclusions

Continuing delays to the implementation of the necessary processes in general practice for the CPRD to be populated demonstrate that mandating a process without first gaining a commitment to implementation on the part of key members of the organisation is highly risky. This is the case even if people agree with the overarching rationale for the actions required. The key problem here is that the CPRD may be presented as a benign, bureaucratic process but the inherent contradictions that are perceived to exist with centrally held values of information governance and consent remains a barrier to implementation.

Although this work is based on general practices in England, it is likely that the conclusions reached about the problems of balancing the contradictions inherent in sharing what can be perceived as a private resource for the public good are globally transferrable.

## Abbreviations

CPRD: Clinical Practice Research Datalink
HRSS: Health Research Support Service
NPT: Normalization process theory
